# Funding of evidence included within public comments submitted to inform Medicare national coverage determinations

**DOI:** 10.1093/haschl/qxae064

**Published:** 2024-05-13

**Authors:** Angela Lu, Robin Z Ji, Marley P D Magee, Joseph S Ross, Reshma Ramachandran, Rita F Redberg, Sanket S Dhruva

**Affiliations:** University of California, San Francisco School of Medicine, San Francisco, San Francisco, CA 94143, United States; Department of Internal Medicine, Mayo Clinic, Rochester, MN 55905, United States; University of California, San Francisco School of Medicine, San Francisco, San Francisco, CA 94143, United States; Pritzker School of Medicine, University of Chicago, Chicago, IL 60637, United States; University of California, Los Angeles, Los Angeles, CA 90095, United States; Section of General Internal Medicine, Department of Internal Medicine, Yale School of Medicine, New Haven, CT 06510, United States; Yale Collaboration for Regulatory Rigor, Integrity, and Transparency (CRRIT), Yale School of Medicine, New Haven, CT 06510, United States; Department of Health Policy and Management, Yale School of Public Health, New Haven, CT 06510, United States; Center for Outcomes Research and Evaluation, Yale New Haven Hospital, New Haven, CT 06510, United States; Section of General Internal Medicine, Department of Internal Medicine, Yale School of Medicine, New Haven, CT 06510, United States; Yale Collaboration for Regulatory Rigor, Integrity, and Transparency (CRRIT), Yale School of Medicine, New Haven, CT 06510, United States; Division of Cardiology, Department of Medicine, University of California, San Francisco School of Medicine, San Francisco, CA 94143, United States; Philip R. Lee Institute for Health Policy Studies, University of California, San Francisco, San Francisco, CA 94143, United States; University of California, San Francisco School of Medicine, San Francisco, San Francisco, CA 94143, United States; Philip R. Lee Institute for Health Policy Studies, University of California, San Francisco, San Francisco, CA 94143, United States; Section of Cardiology, Department of Medicine, San Francisco Veterans Affairs Medical Center, San Francisco, CA 94121, United States

**Keywords:** technology assessment, conflict of interest, disclosure, Medicare

## Abstract

The Centers for Medicare & Medicaid Services (CMS) relies on public comments submitted in response to proposed national coverage determinations to assist the agency in determining the coverage of items and services for Medicare beneficiaries. In a cross-sectional study, we characterized the cited evidence and what funding supported the cited evidence submitted in public comments to CMS for all therapeutic medical device national coverage determinations finalized between June 2019 and June 2022. Of 681 public comments, 159 (23%) cited at least 1 identifiable published scientific journal article. Within these 159 public comments, 198 unique articles were cited, 170 (86%) of which included funding statements or author disclosures. Among these, 96 (56%) disclosed funding from manufacturers that would benefit from Medicare coverage and/or were written by author(s) who received funding from these manufacturers. In summary, most public commenters for national coverage determinations did not cite published scientific journal articles to support their positions. Among those who did, more than half of articles were directly funded by manufacturers that would benefit from coverage. Greater funding of independent, non–industry-supported research may help provide unbiased evaluations of benefits and harms to support Medicare coverage decisions.

## Introduction

The Centers for Medicare & Medicaid Services (CMS) oversees coverage for more than 65 million Medicare beneficiaries.^1^ The CMS is legally bound to cover only items and services that are “reasonable and necessary” for its Medicare beneficiaries.^2^ The CMS may use the national coverage determination (NCD) process, generally when there are significant questions about health outcomes, is new or re-interpreted evidence, is an anticipation of rapid diffusion, or is likely a significant improvement in patient health outcomes or a positive impact on the Medicare program for an item or service.^3^ National coverage determinations are binding decisions that affect all individuals covered by Medicare and supersede any regional coverage determinations. Items and services covered through NCDs must have “sufficient, supporting evidentiary documentation,” including the “relevance, usefulness or the medical benefits” for Medicare beneficiaries.^3^ Medicare NCDs also often influence coverage decisions from other payors.^4^

The NCD process includes multiple opportunities for public comments about draft proposals, allowing external stakeholders to contribute to CMS’ decision-making process prior to NCD finalization; CMS states that public input increases the quality of its decision-making.^5^ The agency is required to consider and respond to all submitted public comments.^3,5^ Public comments citing published clinical evidence have been identified by CMS as being the most helpful.^5^ The agency states that “Public comments that give information on unpublished evidence … are less rigorous and therefore less useful for making a coverage determination.”^5^ The agency has also explained that it historically has based NCDs “on a systematic review of findings reported in the peer-reviewed literature.”^6^

We have previously found that public commenters for NCDs often have undisclosed financial conflicts of interest (COIs) with manufacturers that would benefit from Medicare coverage.^7^ However, little is known about the quality of submitted evidence cited in these comments, if any, and funding of this evidence. Prior research examining public comments submitted to 2 states’ health technology assessment programs found that few comments cited clinical studies.^8^ Comments to CMS about a single NCD infrequently cited scientific literature.^9^ A larger, detailed evaluation of multiple NCDs over a multiyear period is needed to understand what evidence is included within these public comments, the funding sources of the evidence, and if the investigators received funding by manufacturers that would benefit from Medicare coverage.

Medicare NCDs are among the most influential and wide-reaching policy decisions, and often inform the coverage decisions of other public and private payors, in addition to affecting all Medicare beneficiaries. Therefore, it is important to understand if the public comments reference evidence from sources free of financial ties to the coverage decision. Additionally, CMS recently released a procedural notice for a new Transitional Coverage for Emerging Technologies pathway, which will rely on NCDs to inform coverage for a larger number of medical devices.^10,11^ Therefore, considering the patient, clinician, and payer implications of NCDs and CMS’ goal of increasing transparency within the health care system,^12-14^ we systematically analyzed evidence cited in public comments for therapeutic medical devices.

## Data and methods

### National coverage determination process

The NCD process generally encompasses a 9-month period of time, with an initial 30-day public comment period that allows any stakeholder (including patients, physicians, industry, academic institutions, professional societies, and others) to comment about the proposed item or service's risks and benefits to Medicare's beneficiaries.^2^ The CMS then reviews the evidence, considers the public comments, and releases its evaluation and preliminary decision through a proposed NCD memorandum, which is followed by a second 30-day public comment period, after which CMS finalizes the NCD.

### Study design and sample

We conducted a cross-sectional study of all public comments for all therapeutic medical device NCDs finalized between June 2019 and June 2022. These NCDs were for pulmonary embolectomy; transcatheter mitral valve repair (TMVR); artificial hearts and related devices, including ventricular assist devices for bridge-to-transplant and destination therapy; and transcatheter aortic valve replacement (TAVR). Data were collected by 3 investigators (A.L., R.Z.J., M.P.D.M.), with no overlap in data collection. One author (A.L.) reviewed 5% of the data collected by other investigators to ensure consensus. All comments were reviewed manually. If the same commenter commented during both public comment periods for a given NCD, we combined and considered these to be a single comment. Patients were not involved in the design, conduct, or dissemination plans of this research.

### Evidence cited in public comments

To determine the proportion of comments that included supporting evidence, we included any reference to published scientific journal articles: original research studies, guidelines, systematic and non-systematic reviews, as well as commentaries, editorials, perspectives, and viewpoints. The article could have been included as an in-text reference, as a footnote, or as part of the bibliography. If these were not available, we tried to identify the publication by using any available information, including name of a clinical trial, study first author, and/or publication date. Public comments providing clinical evidence without a citation and which we could not locate despite using information available in the comments were classified as “unclear cited evidence.”

### Funding sources in cited evidence

For referenced original research studies and systematic and non-systematic reviews, we determined if the research was directly funded by device manufacturers associated with each NCD. Specifically, we limited this to manufacturers of devices authorized by the Food and Drug Administration (FDA) or at a late stage of development during the public comment periods. For all published scientific journal articles, we also determined whether any study authors had received funding from these device manufacturers by reviewing the disclosure section of the publication.

### Statistical analysis

We used descriptive statistics to summarize data. Institutional review board approval was not required since all data were from publicly available sources.

## Results

### Evidence cited

There were 681 comments for the 4 NCDs, 410 (60%) of which did not cite any published evidence, 112 (16%) cited evidence that could not be located using information in the public comments, and 159 (23%) could be linked to at least 1 published scientific journal article. The proportion of public comments that could be linked to at least 1 published scientific journal article ranged from 12% for the TAVR NCD to 31% for the artificial hearts NCD (Table 1). Overall, 93 (22%) of individual physicians cited evidence (Table 2). Within the 159 public comments that could be linked to at least 1 published scientific journal article, a total of 427 citations were present.

**Table 1.** Characteristics of supporting evidence provided by commenters.

Evidence characteristics of comments	Pulmonary embolectomy	Transcatheter mitral valve repair	Artificial hearts	Transcatheter aortic valve replacement	National coverage determination total
No cited evidence	17 (31%) of 54	186 (61%) of 306	37 (55%) of 68	170 (68%) of 253	410 (60%) of 681
Unclear cited evidence^a^	21 (39%) of 54	27 (9%) of 306	10 (15%) of 68	54 (22%) of 253	112 (16%) of 681
Cited evidence	16 (30%) of 54	93 (30%) of 306	21 (31%) of 68	29 (12%) of 253	159 (23%) of 681
Total number of citations of published journal articles in comments	70	158	50	149	427
Total number of citations of original research studies	49 (70%) of 70	130 (82%) of 158	41 (82%) of 50	111 (74%) of 149	331 (78%) of 427

^a^Public comments categorized as “unclear cited evidence” provided clinical evidence that we could not locate despite using all available information in the text of the comment.

**Table 2.** Proportion of commenters citing evidence in public comments for Medicare national coverage determinations.

	Did not cite, *n* (%)	Unclear citation, *n* (%)	Cited, *n* (%)
Individuals			
Physicians	271 (64)	57 (14)	93 (22)
Group of physicians	0 (0)	3 (50)	3 (50)
Non-physician health care professional	45 (65)	15 (22)	9 (13)
Patient or family member	30 (61)	12 (25)	7 (14)
Other	3 (100)	0 (0)	0 (0)
Organizations			
Hospital or health systems	37 (58)	12 (19)	15 (23)
Device manufacturer with relevant device(s) of interest in NCD	6 (46)	1 (8)	6 (46)
Other device manufacturer	3 (38)	3 (38)	2 (25)
Device trade organization	1 (20)	1 (20)	3 (60)
Insurer	0 (0)	1 (100)	0 (0)
Professional society	5 (21)	5 (21)	14 (58)
Other organization^a^	9 (50)	2 (11)	7 (39)

Abbreviation: NCD, national coverage determination.

^a^Other organizations included nonprofit organizations, nondevice manufacturers or trade organizations, consulting or advisory firms, and health policy centers.

Of the 427 total citations, there were 198 unique published scientific journal articles; this ranged from 29 for pulmonary embolectomy to 82 for TAVR (Table 3). Approximately three-fourths (142; 72%) of these unique articles were original research studies, 18 (9%) were clinical practice guidelines, 29 (15%) were reviews, and 9 (5%) were commentaries, editorials, perspectives, or viewpoints.

**Table 3.** Characteristics of published scientific journal articles cited in public comments about national coverage determinations of therapeutic medical devices from June 2019 through June 2022.

Scientific journal article characteristics	Pulmonary embolectomy	Transcatheter mitral valve repair	Artificial hearts	Transcatheter aortic valve replacement	Total, *n* (%)
Unique published articles cited in comments	29	50	37	82	198
Unique original research studies	18 (62%) of 29	36 (72%) of 50	28 (76%) of 37	60 (73%) of 82	142 (72%) of 198
Unique guidelines	3 (10%) of 29	7 (14%) of 50	1 (3%) of 37	7 (9%) of 82	18 (9%) of 198
Unique reviews	7 (24%) of 29	4 (8%) of 50	5 (14%) of 37	13 (16%) of 82	29 (15%) of 198
Unique commentaries, editorials, perspectives, or viewpoints	1 (3%) of 29	3 (6%) of 50	3 (8%) of 37	2 (2%) of 82	9 (5%) of 198

### Funding of published evidence cited in public comments

Of the 198 unique published scientific journal articles that were cited in public comments, 170 included funding or author disclosures within the manuscript (Table 4). Of these 170, 96 (56%) publications had either direct funding for the study and/or at least 1 author who received funding from a device manufacturer that would benefit from expanded device coverage, ranging from 44% of publications for the pulmonary embolectomy NCD to 69% for the TMVR NCD.

**Table 4.** Funding of published scientific journal articles cited in public comments about national coverage determinations of therapeutic medical devices from June 2019 through June 2022.

Scientific journal article characteristics	Pulmonary embolectomy	Transcatheter mitral valve repair	Artificial hearts	Transcatheter aortic valve replacement	Total, *n* (%)
Device manufacturer funding or author financial support in article					
Article funding and author disclosures could be found	25 (86%) of 29	42 (84%) of 50	34 (92%) of 37	69 (84%) of 82	170 (86%) of 198
Manufacturer involvement present for article or at least 1 author	11 (44%) of 25	29 (69%) of 42	18 (53%) of 34	38 (55%) of 69	96 (56%) of 170
Author disclosures					
Author disclosures could not be found	5 (17%) of 29	14 (28%) of 50	3 (8%) of 37	21 (26%) of 82	43 (22%) of 198
At least 1 author had received funding from manufacturers	11 (46%) of 24	27 (75%) of 36	17 (50%) of 34	37 (61%) of 61	92 (59%) of 155
Manufacturer funding of original research^a^					
Funding could not be found	14 (56%) of 25	25 (63%) of 40	15 (45%) of 33	32 (44%) of 73	86 (50%) of 171
Direct manufacturer funding	4 (36%) of 11	10 (67%) of 15	6 (33%) of 18	16 (39%) of 41	36 (42%) of 85

Manufacturers of devices included manufacturers who had devices authorized by the Food and Drug Administration (FDA) or at a late stage of development during the public comment periods.

^a^As guidelines and commentaries, editorials, perspectives, and viewpoints do not receive direct manufacturer funding, they were excluded from manufacturer funding review.

Of the 198 unique publications, disclosures were not included for 43, leaving 155 publications with author disclosures (Table 4). Of these 155 publications, 92 (59%) had at least 1 author who had received funding from a relevant device manufacturer. The proportion of unique publications with author funding from relevant device manufacturers ranged from 46% for the pulmonary embolectomy NCD to 75% for the TMVR NCD.

After excluding guidelines, commentaries, editorials, perspectives, and viewpoints, 171 original research studies or reviews were included. Of these 171, funding information was unavailable for 86 (50%). Among the 85 (50%) with funding information available, 36 (42%) were directly funded by device manufacturers that would benefit from expanded device coverage. This ranged from 33% for the artificial hearts NCD to 67% for the TMVR NCD.

## Discussion

In this manual review of all public comments to CMS for 4 medical device NCDs from 2019 to 2022, most (60%) public comments did not cite any published scientific journal articles. This gap represents an opportunity to provide more useful information in the form of clinical evidence for CMS from public comments to better assist the agency in finalizing the NCD. Furthermore, when evidence was cited and funding or disclosures were available, most articles were either directly funded by the manufacturer or included at least 1 author who disclosed funding from a device manufacturer that would benefit from Medicare coverage. The CMS staff are sophisticated reviewers with experience reviewing and considering public comments. However, given that industry-funded research is more likely to have positive, pro-industry conclusions,^15,16^ our findings have implications about a source of possible industry influence on coverage decisions by the United States’ largest national insurer.

Although CMS emphasizes that its evaluation of comments places greater importance on clinical evidence rather than anecdotes of personal experience,^3,5^ most commenters did not cite published scientific journal articles, consistent with a previous study of a single NCD.^9^ Prior research has found that Medicare coverage was associated with consistent favorable clinical evidence and guideline recommendations.^17^ Therefore, commenters would benefit from citing peer-reviewed evidence, especially studies that evaluate benefits and harms among populations representative of Medicare beneficiaries. More consistent guidance from CMS about how to include evidence as part of public comments, such as a templated section for citing evidence in the comment submission system, could help. The CMS has also recently published guidance about the type of evidence that would best support coverage determinations for medical devices.^10,18^ While the agency reviews available evidence for its NCDs through internal or external technology assessments, commenters may identify studies that are not identified and may provide informative perspectives about the published evidence.

Most (56%) published research articles that were cited as evidence in public comments were found to have funding from device manufacturers or included an author with a disclosure from device manufacturers that would benefit financially from coverage. The actual percentage of publications with ties to the manufacturers is likely even higher, as 80% of authors of drug trial manuscripts did not disclose industry payments.^19^ Industry-funded studies are more likely to demonstrate favorable efficacy results.^15,16,20-24^ Further, author financial COIs are also associated with reporting of positive findings.^25,26^ Although almost all premarket device studies are industry-funded,^27^ since industry is incentivized to study their devices, we studied NCDs for devices that were many years post–FDA approval, including artificial hearts and TAVR. There are important mechanisms to ensure the trustworthiness of research, including registration and reporting of clinical trials at ClinicalTrials.gov. Although this has significantly improved registration and publication,^28^ results are not always reported and, when reported,^29^ there are often discrepancies and selective reporting of endpoints.^30^ Further, registration and reporting are not required for observational studies.^31^ Additional congressional funding of independent comparative effectiveness studies of medical devices, such as through the Patient Centered Outcomes Research Institute, could generate more unbiased evidence, particularly given the increased dependence on NCDs in CMS' Transitional Coverage for Emerging Technologies pathway.^10,11^ There is also the opportunity to leverage real-world electronic health record and claims data, to support both the conduct of pragmatic randomized controlled trials^32^ and rigorous observational studies that may also better detect rare outcomes.^33^

Our study is limited to 4 NCDs for therapeutic medical devices, and findings may not generalize to other classes of services considered by CMS. Additionally, we considered only funding reported by authors; as research has demonstrated underreporting of COIs,^19,26,34^ it is likely that our findings underestimate the extent to which evidence included within comments submitted to CMS is conducted by authors with funding from manufacturers that would benefit from Medicare coverage.

## Conclusion

Most public comments for Medicare NCDs do not include supporting evidence in the form of published scientific journal articles. When evidence is provided and funding or disclosure information was available, most of the cited evidence has been directly funded by and/or written by author(s) who have received funding from manufacturers that would benefit from Medicare coverage. Greater funding of independent, non–industry-supported research may help provide unbiased evaluations of benefits and harms of items and services to support coverage decisions for Medicare beneficiaries.

## Supplementary Material

qxae064_Supplementary_Data
